# Global Reproducibility Through Local Control for Distributed Active Objects

**DOI:** 10.1007/978-3-030-45234-6_7

**Published:** 2020-03-13

**Authors:** Lars Tveito, Einar Broch Johnsen, Rudolf Schlatte

**Affiliations:** 8 University of Paderborn, Paderborn, Germany; 9 ICREA, Open University of Catalonia, Barcelona, Spain; Department of Informatics, University of Oslo, P.O. box 1080, Blindern, 0316 Oslo, Norway

## Abstract

Non-determinism in a concurrent or distributed setting may lead to many different runs or executions of a program. This paper presents a method to reproduce a specific run for non-deterministic actor or active object systems. The method is based on recording traces of events reflecting local transitions at so-called stable states during execution; i.e., states in which local execution depends on interaction with the environment. The paper formalizes trace recording and replay for a basic active object language, to show that such local traces suffice to obtain global reproducibility of runs; during replay different objects may operate fairly independently of each other and in parallel, yet a program under replay has guaranteed deterministic outcome. We then show that the method extends to the other forms of non-determinism as found in richer active object languages. Following the proposed method, we have implemented a tool to record and replay runs, and to visualize the communication and scheduling decisions of a recorded run, for Real-Time ABS, a formally defined, rich active object language for modeling timed, resource-aware behavior in distributed systems.

### 1 Introduction

Non-determinism in a concurrent or distributed setting leads to many different possible runs or executions of a given program. The ability to reproduce and visualize a particular run can be very useful for the developer of such programs. For example, reproducing a specific run representing negative (or unexpected) behavior can be beneficial to eliminate bugs which occur only in a few out of many possible runs (so-called Heisenbugs). Conversely, reproducing a run representing positive (and expected) behavior can be useful for regression testing for new versions of a system.

Deterministic replay is an emerging technique to provide deterministic executions of programs in the presence of different non-deterministic factors [1]. In a first phase, the technique consists of *recording* sufficient information in a trace during a run to reproduce the same run during a *replay* in a second phase. Approaches to reproduce runs of non-deterministic systems can be classified as either *content-based* or *ordering-based* replay. Content-based replay records the results of all non-deterministic operations whereas ordering-based replay records the ordering of non-deterministic events.

This paper considers deterministic replay for non-deterministic runs of Active Object languages [2], which combine the asynchronous message passing of Actors with object-oriented abstractions. Compared to standard OO languages, these languages decouple communication and synchronization by communicating through asynchronous method calls without transfer of control and by synchronizing via futures. We develop a method to reproduce the runs of active objects. The method is ordering-based, as we represent the parallel execution of active objects as traces of events. We show that locally recording events at so-called *stable states* suffice to obtain deterministic replay. In these states, local execution needs to interact with the environment, e.g., to make a scheduling decision or to send or receive a message. We formalize execution with record and replay for a basic active object language, and show that its executions enjoy confluence properties which can be described using such traces. These confluence properties justify the recording and replay of local traces to reproduce global behavior.

Active object languages may also contain more advanced features [2], such as *cooperative scheduling* [3,4], *concurrent object groups* [3,5] and *timed, resource-aware behavior* [6]. With cooperative scheduling, an object may suspend its current task while waiting for the result of a method call and instead schedule a different task. With concurrent object groups, several objects share an actor's lock abstraction. With timed, resource-aware behavior, local execution requires resources from resource-centers (e.g., virtual machines) to progress. These features introduce additional non-determinism in the active object systems, in addition to the non-determinism caused by asynchronous calls. We show that the proposed method extends to handle these additional sources of non-determinism.

The proposed method to deterministically replay runs has been realized for Real-Time ABS [6], a modeling language with these advanced features, which has been used to analyze, e.g., industrial scale cloud-deployed software [7], railway networks [8], and complex low-level multicore systems [9,10]. Whereas the language supports various formal analysis techniques, most validation of complex models (at least in an early stage of model development) is based on simulation. The tracing capabilities have a small enough performance impact to be enabled by default in the simulator. The simulator itself is implemented as a distributed system in Erlang [11]. The low performance overhead comes from only recording local events in each actor, which does not impose any additional communication or synchronization, which are typically bottlenecks in a distributed system.

*Contributions.* Our main contributions can be summarized as follows:

– we propose a method to reproduce runs for active object systems based on recording events reflecting local transitions from stable object states;
– we provide a formal justification for the method in terms of confluence and progress properties for ordering-based record & replay for a basic actor language with asynchronous communication and synchronization via futures;
– we show that the method extends to address additional sources of non-determinism as found in richer active object languages; and
– we provide an implementation of the proposed method to record, replay and visualize runs for the active object modeling language Real-Time ABS.

```
class C {
  Int n = 1;
  Unit m1() { n = n - 1; }
  Unit m2() { n = n * 3; }
}

// Main block
{
  C o = new C();
  o!m1();
  o!m2();
}
```

Fig. 1: A simple program, with two possible results

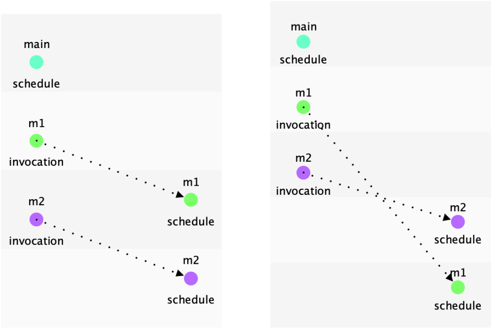

Fig. 2: The executions leading to the two different results for the simple program.

*Paper overview* Section 2 provides a motivating example, Section 3 considers the problem of reproducibility for a formalization of a basic active object language and Section 4 formalizes record and replay over the operational semantics of the basic language. Section 5 considers reproducibility for extensions to the basic language. Section 6 presents our implementation of the method for Real-Time ABS. Section 7 discusses related work and Section 8 concludes the paper.

### 2 Motivating Example

Consider the program in Fig. 1. It consists of a class `C`, with a single integer field, initialized to 1 and two methods `m1` and `m2`. The main block of the program creates an active object *o* as an instance of the class, and performs two asynchronous calls on *o*, `o!m1()` and `o!m2()` respectively. Since the calls are asynchronous, the caller can proceed to make the second call immediately, without waiting for the first call to complete. The two calls are placed in the queue of *o* and scheduled in some order for execution by *o*. (We here assume method execution is atomic, but this assumption will be relaxed in Section 5.)

Thus, even the execution of this very simple program can lead to two different results, depending on whether `o!m1()` is scheduled before `o!m2()`, and conversely, `o!m2()` is scheduled before `o!m1()`. In the first case, the field `n` (which is initially 1) will first be decremented by 1 and then be multiplied with 3, resulting in a final state in which the field `n` has the value 0. In the second case, the field `n` is first multiplied by 3, then decremented by 1, resulting in a final state in which the field `n` has the value 2. Fig. 2 depicts the two cases (using the visualization support in our tool, described in Section 6.3). Note that this problem still occurs for languages with ordered message passing between two actors (e.g., Erlang [11]) when the two calls are made by different callers.

The selection of run to execute is decided by the runtime system and is thus non-deterministic for the given source program. In general, there can be much more than two possible runs for a parallel active object system. If only a few of the possible runs exhibit a particular behavior (e.g., a bug), it can be very interesting to be able to reproduce a particular run of the given program. We propose a method to instrument active objects systems which allows global reproducibility of runs through local control for each active object.

### 3 A Formal Model of Reproducibility

To formalize the problem of global reproducibility through local control for active object systems, we consider a basic active object language in which non-determinism stems from the order in which method calls are selected from the queue of the active objects.

#### 3.1 A Basic Active Object Language

Consider a basic active object language with asynchronous method calls and synchronization via futures. The language has a Java-like syntax, given in Fig. 3. Let $T$, $C$ and $m$ range over type, class and method names, respectively, and let $e$ range over side-effect free expressions. Overlined terms denote possibly empty lists over the corresponding syntactic categories (e.g., $\overline{e}$ and $\overline{x}$).

$$
\begin{aligned}
P &::= \overline{CL}\ \{\overline{T\ x};\ s\} \\
CL &::= \texttt{class}\ C\,\{\overline{T\ x};\ \overline{M}\} \\
M &::= T\ m\ (\overline{T\ x})\,\{\overline{T\ x};\ s\} \\
s &::= s; s \mid \texttt{skip} \mid x = rhs \\
&\quad \mid \texttt{if}\ e\ \{s\}\,\texttt{else}\,\{s\} \\
&\quad \mid \texttt{while}\ e\,\{s\} \mid \texttt{return}\ e \\
rhs &::= e \mid \texttt{new}\ C\,(\overline{e}) \mid e!m(\overline{e}) \mid x.\texttt{get}
\end{aligned}
$$

Fig. 3: BNF for the basic active object language.

A program $P$ consists of a list $\overline{CL}$ of class declarations and a main block $\{\overline{T\ x}; s\}$, with variables $x$ of type $T$ and a statement $s$. A class $C$ declares fields (both with types $T$) and contains a list $\overline{M}$ of methods. A method $m$ has a return type, a list of typed formal parameters and a method body which contains local variable declarations and a statement $s$. Statements are standard; assignments $x = rhs$ allow expressions with side-effects on the right-hand side $rhs$.

Asynchronous method calls decouple invocation from synchronization. The execution of a call `f = o!m(args)` corresponds to sending a message `m(args)` asynchronously to the callee object $o$ and initializes a future, referenced by `f`, where the return value will be stored. The statement `x = f.get` retrieves the value stored in the future `f`. This operation synchronizes with the method return; i.e., the execution of this statement *blocks* the active object until the future `f` has received a value. Messages are *not* assumed to arrive in the same order as they are sent. The selection of messages in an object gives rise to non-determinism in the execution. An example of a program in this language was given in Section 2.

#### 3.2 An Operational Semantics for the Basic Language

We present the semantics of the basic active object language as a transition relation between configurations $cn$. In the runtime syntax (Fig. 4), a configuration $cn$ can be empty ($\epsilon$), or a set of objects, futures, and invocation messages. We let $o$ and $f$ be dynamically created names from a set of object and future

$$\begin{aligned} cn &::= \epsilon \mid object \mid future \mid invoc \mid cn\ cn & q &::= \epsilon \mid process \mid q\ q \\ future &::= fut(f, val) & val &::= v \mid \bot \\ object &::= ob(o, a, p, q) & a &::= x \mapsto v \mid a, a \\ process &::= \{a \mid s\} & p &::= process \mid \texttt{idle} \\ invoc &::= inv(o, f, m, \overline{v}) & v &::= o \mid f \mid \texttt{true} \mid \texttt{false} \mid t \end{aligned}$$

Fig. 4: Runtime syntax; here, $o$ and $f$ are object and future identifiers.

identifiers, denoted *Identifiers*. An active object $ob(o, a, p, q)$ has an identifier $o$, attributes $a$, an active process $p$ (that may be `idle`) and an unordered process pool $q$. A future $fut(f, val)$ has an identifier $f$ and a value $val$ (which is $\bot$ if the future is not resolved). An invocation $inv(o, f, m, \overline{v})$ is a message to object $o$ to activate method $m$ with actual parameters $\overline{v}$ and send the return value to the future $f$. Attributes bind program variables $x$ to values $v$. A process $\{a \mid s\}$ has local variables $a$ and a statement list $s$ to execute. Values are object identifiers $o$, future identifiers $f$, Boolean values `true` and `false`, and other literal values $t$ (e.g., natural numbers). The initial state of a program consists of a single active objects $ob(o_{\texttt{main}}, a, p, \emptyset)$, where the active process $p$ corresponds to the main block of the program. Let $names(cn)$ denote the set of object and future identifiers occurring in a configuration $cn$.

Figure 5 presents the main rules of the transition relation $cn \to cn'$. A *run* is a finite sequence of configurations $cn_0, cn_1, \ldots, cn_n$ such that $cn_i \to cn_{i+1}$ for $0 \leqslant i < n$. We assume configurations to be associative and commutative (so we can reorder configurations to match rules), where $\xrightarrow{*}$ denotes the reflexive and transitive closure of $\to$. Let $bind(m, \overline{v}, f, C)$ denote method lookup in the class table, returning the process corresponding to method $m$ in class $C$ with actual parameters $\overline{v}$ and with future $f$ as the return address of the call. Thus, every process has a local variable *destiny* which denotes the return address of the process (i.e., the future that the process will resolve upon completion), similar to the self-reference *this* for objects. We omit explanations for the standard rules for assignment to fields and local variables, conditionals, while and skip.

Rule Activate formalizes the scheduling of a process $p$ from the unordered queue $q$ when an active object is idle. In Async-Call, an asynchronous method call creates a message to a target object $o'$ and an unresolved future with a fresh name $f$. Object creation in New-Actor creates a new active object with a fresh identifier $o'$, and initializes its attributes with $\text{initAttributes}(C, o')$, including reference to itself (`this`). These are the only rules that introduce new names for identifiers; let a predicate $\text{fresh}(o)$ denote that $o$ is a fresh name in the global configuration (abstracting from how this is implemented). Rule Load puts the process corresponding to an invocation message in called object's queue. Rule Return resolves the future associated with a process with return value $v$, and Read-Fut fetches the value $v$ of a future $f$ into a variable. With rule Context, parallel execution in different active objects has an interleaving semantics.

**Definition 1 (Stable configurations).** *A configuration cn is* stable *if, for all objects in cn, the execution is blocked or the object needs to make a scheduling*

$$\frac{p \in q}{ob(o,a,\texttt{idle},q) \rightarrow ob(o,a,p,q\backslash\{p\})}\ (\textsc{Activate})$$

$$\frac{v = [\![e]\!]_{(a\circ l)} \quad x \in \mathrm{dom}(l)}{ob(o,a,\{l \mid x = e; s\},q) \rightarrow ob(o,a,\{l[x \mapsto v] \mid s\},q)}\ (\textsc{Assign1})$$

$$\frac{v = [\![e]\!]_{(a\circ l)} \quad x \notin \mathrm{dom}(l)}{ob(o,a,\{l \mid x = e; s\},q) \rightarrow ob(o,a[x \mapsto v],\{l \mid s\},q)}\ (\textsc{Assign2})$$

$$\frac{\texttt{true} = [\![e]\!]_{(a\circ l)}}{ob(o,a,\{l \mid \texttt{if}\ e\ \{s_1\}\ \texttt{else}\ \{s_2\}; s\},q) \rightarrow ob(o,a,\{l \mid s_1; s\},q)}\ (\textsc{Cond1})$$

$$\frac{\texttt{false} = [\![e]\!]_{(a\circ l)}}{ob(o,a,\{l \mid \texttt{if}\ e\ \{s_1\}\ \texttt{else}\ \{s_2\}; s\},q) \rightarrow ob(o,a,\{l \mid s_2; s\},q)}\ (\textsc{Cond2})$$

$$\frac{s_1' = s_1; \texttt{while}\ e\ \{s_1\}}{ob(o,a,\{l \mid \texttt{while}\ e\ \{s_1\}; s_2\},q) \rightarrow ob(o,a,\{l \mid \texttt{if}\ e\ \{s_1'\}\ \texttt{else}\ \{\texttt{skip}\}; s_2\},q)}\ (\textsc{While})$$

$$(\textsc{Skip1})\quad ob(o,a,\{l \mid \texttt{skip}; s\},q) \rightarrow ob(o,a,\{l \mid s\},q)$$

$$(\textsc{Skip2})\quad ob(o,a,\{l \mid \texttt{skip}\},q) \rightarrow ob(o,a,\texttt{idle},q)$$

$$\frac{a' = \mathrm{initAttributes}(C,o') \quad \mathrm{fresh}(o')}{ob(o,a,\{l \mid x = \texttt{new}\ C(); s\},q) \rightarrow ob(o,a,\{l \mid x = o'; s\},q)\ ob(o',a',\texttt{idle},\emptyset)}\ (\textsc{New-Actor})$$

$$\frac{cn_1 \rightarrow cn_1'}{cn_1\ cn_2 \rightarrow cn_1'\ cn_2}\ (\textsc{Context})$$

$$\frac{o' = [\![e]\!]_{(a\circ l)} \quad \overline{v} = [\![\overline{e}]\!]_{(a\circ l)} \quad \mathrm{fresh}(f)}{ob(o,a,\{l \mid x = e!m(\overline{e}); s\},q) \rightarrow ob(o,a,\{l \mid x = f; s\},q)\ inv(o',f,m,\overline{v})\ fut(f,\bot)}\ (\textsc{Async-Call})$$

$$\frac{p' = \mathrm{bind}(m,\overline{v},f,\mathrm{classOf}(o))}{inv(o,f,m,\overline{v})\ ob(o,a,p,q) \rightarrow ob(o,a,p,q \cup \{p'\})}\ (\textsc{Load})$$

$$\frac{v = [\![e]\!]_{(a\circ l)} \quad l(\mathrm{destiny}) = f}{ob(o,a,\{l \mid \texttt{return}\ e\},q)\ fut(f,\bot) \rightarrow ob(o,a,\texttt{idle},q)\ fut(f,v)}\ (\textsc{Return})$$

$$\frac{v \neq \bot \quad f = [\![e]\!]_{(a\circ l)}}{ob(o,a,\{l \mid x = e.\texttt{get}; s\},q)\ fut(f,v) \rightarrow ob(o,a,\{l \mid x = v; s\},q)\ fut(f,v)}\ (\textsc{Read-Fut})$$

Fig. 5: Semantics of the basic active object language.

*decision. An object is blocked if it needs to execute a get-statement. An object needs to make a scheduling decision if its active process is idle.*

Let $G$ denote a stable configuration. We say that two stable configurations $G_1$ and $G_2$ are *consecutive* in a run $G_1 \xrightarrow{*} G_2$ if, for all $cn$ such that $G_1 \xrightarrow{*} cn$ and $cn \xrightarrow{*} G_2$, if $cn \neq G_1$ and $cn \neq G_2$ then $cn$ is not a stable configuration.

**Lemma 1 (Reordering of atomic sections).** *Let $G_1$ and $G_2$ be stable configurations. If $G_1 \xrightarrow{*} G_2$, then there exists a run between $G_1$ and $G_2$ in which only a single object executes between any two consecutive stable configurations.*

*Proof (sketch).* Observe that the notion of stability captures any state of an object in which it needs input from its environment. The proof then follows from the fact that the state spaces of different objects are disjoint and that message passing is unordered. This allows consecutive independent execution steps from different objects to be reordered. □

$$\text{(Local-Assign1)}\ \frac{v = [\![e]\!]_{(a\circ l)} \quad x \in \mathrm{dom}(l)}{a, \{l \mid x = e; s\} \rightsquigarrow a, \{l[x \mapsto v] \mid s\}} \qquad \text{(Local-Assign2)}\ \frac{v = [\![e]\!]_{(a\circ l)} \quad x \notin \mathrm{dom}(l)}{a, \{l \mid x = e; s\} \rightsquigarrow a[x \mapsto v], \{l \mid s\}}$$

$$\text{(Local-While)}\ \frac{s_1' = s_1; \texttt{while}\ e\ \{s_1\}}{a, \{l \mid \texttt{while}\ e\ \{s_1\}; s_2\} \rightsquigarrow a, \{l \mid \texttt{if}\ e\ \{s_1'\}\ \texttt{else}\ \{\texttt{skip}\}; s_2\}} \qquad \text{(Local-Skip1)}\ a, \{l \mid \texttt{skip}; s\} \rightsquigarrow a, \{l \mid s\}$$

$$\text{(Local-Cond1)}\ \frac{\texttt{true} = [\![e]\!]_{(a\circ l)}}{\begin{array}{c}a, \{l \mid \texttt{if}\ e\ \{s_1\}\ \texttt{else}\ \{s_2\}; s\}\\ \rightsquigarrow a, \{l \mid s_1; s\}\end{array}} \qquad \text{(Local-Cond2)}\ \frac{\texttt{false} = [\![e]\!]_{(a\circ l)}}{\begin{array}{c}a, \{l \mid \texttt{if}\ e\ \{s_1\}\ \texttt{else}\ \{s_2\}; s\}\\ \rightsquigarrow a, \{l \mid s_2; s\}\end{array}} \qquad \text{(Local-Skip2)}\ a, \{l \mid \texttt{skip}\} \rightsquigarrow a, \texttt{idle}$$

$$\text{(Global-Activate)}\ \frac{\begin{array}{c}p \in q \quad a, p \overset{!}{\rightsquigarrow} a', p' \quad p = \{l \mid s\}\\ l(\text{destiny}) = f \quad q' = q \backslash \{p\}\end{array}}{\begin{array}{c}ob(o, a, \texttt{idle}, q)\\ \xrightarrow{sched\langle o,f\rangle} ob(o, a', p', q')\end{array}} \qquad \text{(Global-Return)}\ \frac{v = [\![e]\!]_{(a\circ l)} \quad l(\text{destiny}) = f}{\begin{array}{c}ob(o, a, \{l \mid \texttt{return}\ e\}, q)\ fut(f, \bot)\\ \xrightarrow{futWr\langle o,f\rangle} ob(o, a, \texttt{idle}, q)\ fut(f, v)\end{array}} \qquad \text{(Global-Context)}\ \frac{cn_1 \xrightarrow{ev?} cn_1'}{\begin{array}{c}cn_1\ cn_2\\ \xrightarrow{ev?} cn_1'\ cn_2\end{array}}$$

$$\text{(Global-New-Actor)}\ \frac{\begin{array}{c}a'' = \mathrm{initAttributes}(C, o')\\ \mathrm{fresh}(o') \quad a, \{l \mid x = o'; s\} \rightsquigarrow a', p'\end{array}}{\begin{array}{c}ob(o, a, \{l \mid x = \texttt{new}\ C(); s\}, q)\\ \xrightarrow{new\langle o,o'\rangle} ob(o, a', p', q)\ ob(o', a'', \texttt{idle}, \emptyset)\end{array}} \qquad \text{(Global-Read-Fut)}\ \frac{\begin{array}{c}v \neq \bot \quad f = [\![e]\!]_{(a\circ l)}\\ a, \{l \mid x = v; s\} \overset{!}{\rightsquigarrow} a', p\end{array}}{\begin{array}{c}ob(o, a, \{l \mid x = e.\texttt{get}; s\}, q)\ fut(f, v)\\ \xrightarrow{futRe\langle o,f\rangle} ob(o, a', p, q)\ fut(f, v)\end{array}}$$

$$\text{(Global-Async-Call)}\ \frac{\begin{array}{c}o' = [\![e]\!]_{(a\circ l)} \quad \overline{v} = [\![\overline{e}]\!]_{(a\circ l)}\\ \mathrm{fresh}(f) \quad a, \{l \mid x = f; s\} \overset{!}{\rightsquigarrow} a', p\end{array}}{\begin{array}{c}ob(o, a, \{l \mid x = e!m(\overline{e}); s\}, q)\\ \xrightarrow{inv\langle o,f\rangle} ob(o, a', p, q)\ inv(o', f, m, \overline{v})\ fut(f, \bot)\end{array}} \qquad \text{(Global-Load)}\ \frac{p' = \mathrm{bind}(m, \overline{v}, f, \mathrm{classOf}(o))}{\begin{array}{c}inv(o, f, m, \overline{v})\ ob(o, a, p, q)\\ \rightarrow ob(o, a, p, q \cup \{p'\})\end{array}}$$

Fig. 6: Coarse-grained, labelled semantics of the basic active object language.

#### 3.3 A Labelled Operational Semantics for the Basic Language

Based on Lemma 1, we can define a semantics of the basic active object language with a more coarse-grained model of interleaving which is equivalent to the semantics presented in Fig. 5. We let this coarse-grained semantics be labeled by events to record the interaction between an active object and its environment. The events are defined as follows:

**Definition 2 (Events).** *Let $o,f \in Identifiers$. The set $\mathcal{E}$ of* events *$ev$ is given by*

$$ev ::= new\langle o, o\rangle \mid inv\langle o, f\rangle \mid sched\langle o, f\rangle \mid futWr\langle o, f\rangle \mid futRe\langle o, f\rangle.$$

In the coarse-grained semantics, a transition relation $a, p \rightsquigarrow a', p'$ captures *local execution* in an active object with attributes $a$. These rules are given in

Fig. 6 (top) and correspond to the rules Assign1, Assign2, While, Cond1, Cond2, Skip1 and Skip2 of Fig. 5. These rules are deterministic as there is at most one possible reduction for any given pair $a, p$. Let $\overset{*}{\rightsquigarrow}$ denote the reflexive, transitive closure of $\rightsquigarrow$, let the unary relation $\not\rightsquigarrow$ denote that there is no transition from a given pair $a, p$, and let the relation $\overset{!}{\rightsquigarrow}$ denote the reduction to normal form according to $\rightsquigarrow$; i.e.,

$$a, p \overset{!}{\rightsquigarrow} a', p' \iff a, p \overset{*}{\rightsquigarrow} a', p' \wedge a', p' \not\rightsquigarrow$$

In the remaining rules, given in Fig. 6 (bottom), a labelled transition relation $cn \xrightarrow{ev} cn'$ captures transitions in which the local execution of an active object interacts with its environment through scheduling, object creation, method invocation, or interaction with futures. These rules also correspond to the similar rules in Fig. 5, with two differences:

1. The rules are labelled with an event reflecting the particular action taken in the transition, and
2. the rules perform a local deterministic reduction to normal form according to the $\rightsquigarrow$ relation in each step.

Remark that rule Global-Load is identical to Load of Fig. 5; although we do not need to add an explicit label the rule is kept at the global level since it involves both an object and a message. Rule Global-Context is labeled by $ev?$ to capture that the label is optional (i.e., the rule also combines with Global-Load). We henceforth consider runs for the basic active object language based on this labelled semantics.

#### 3.4 Execution Traces and their Reordering

This section looks at traces reflecting the runs of programs in the basic active object language according to the semantics of Section 3.3, and their reordering. We consider traces over events in $\mathcal{E}$. Let $\epsilon$ denote the empty trace, and $\tau_1 \cdot \tau_2$ the concatenation of traces $\tau_1$ and $\tau_2$. For an event $ev$ and a trace $\tau$, we denote by $ev \in \tau$ that $ev$ occurs somewhere in $\tau$ and by $\tau$ **ew** $ev$ that $\tau$ ends with $ev$ (i.e., $\exists \tau'.\tau = \tau' \cdot ev$). Define $\tau/o$ and $\tau/f$ as the projection of a trace $\tau$ to the alphabet of an object $o$ and a future $f$, by their first or second argument respectively (where an alphabet is the set of events involving that name). Finally, let $names(\tau)$ denote the inductively defined function returning the set of identifiers that occur in a trace $\tau$ (e.g., $names(inv\langle o, f\rangle) = \{o, f\}$). We assume that every initial configuration has a main object and process, and let $names(\epsilon) = \{o_{\mathtt{main}}, f_{\mathtt{main}}\}$.

Given a run $cn_0 \xrightarrow{ev_0} \cdots \xrightarrow{ev_n} cn_{n+1}$, we denote $cn_0 \overset{\tau}{\Rightarrow} cn_{n+1}$ that a trace $\tau$ is the trace of the run if $\tau = ev_0 \cdots ev_n$ (where $\tau$ ignores the unlabeled transition steps of the run). Well-formed traces can be defined as follows, based on [12]:

**Definition 3 (Well-formed Traces).** *Given* $o, o', f \in \mathit{Identifiers}$. *Let* $\mathit{wf}(\tau)$ *denote that* $\tau$ *is* well-formed, *defined inductively:*

$$\begin{aligned}
&\mathit{wf}(\epsilon) \iff \mathit{True}\\
&\mathit{wf}(\tau \cdot \mathit{new}\langle o, o'\rangle) \iff \mathit{wf}(\tau) \wedge o \in \mathit{names}(\tau) \wedge o' \notin \mathit{names}(\tau)\\
&\mathit{wf}(\tau \cdot \mathit{inv}\langle o, f\rangle) \iff \mathit{wf}(\tau) \wedge o \in \mathit{names}(\tau) \wedge f \notin \mathit{names}(\tau)\\
&\mathit{wf}(\tau \cdot \mathit{sched}\langle o, f\rangle) \iff \mathit{wf}(\tau) \wedge o \in \mathit{names}(\tau) \wedge \tau/f = \mathit{inv}\langle o', f\rangle\\
&\mathit{wf}(\tau \cdot \mathit{futWr}\langle o, f\rangle) \iff \mathit{wf}(\tau) \wedge \tau/f \ \mathbf{ew}\ \mathit{sched}\langle o, f\rangle\\
&\mathit{wf}(\tau \cdot \mathit{futRe}\langle o, f\rangle) \iff \mathit{wf}(\tau) \wedge \mathit{futWr}\langle o', f\rangle \in \tau
\end{aligned}$$

Wellformedness thus captures a happens-before relation over events while ensuring that certain identifiers are new at given points in the trace. Din and Owe have shown that the trace of any run of the semantics of an active object language similar to ours is well-formed [12]. For example, no process can be scheduled unless it has been invoked (which again requires the GLOBAL-LOAD rule to apply in between GLOBAL-ASYNC-CALL and GLOBAL-ACTIVATE). Given a trace $\tau$, we can now define the equivalence class $[\tau]$ of traces which preserve the local ordering and the wellformedness of $\tau$, as follows:

**Definition 4 (Global trace set).** *Let* $\tau$ *be a trace and define*

$$[\tau] = \{\tau' \mid \tau'/o = \tau/o \text{ for all object identifiers } o \in \mathit{names}(\tau) \wedge \mathit{wf}(\tau')\}.$$

Remark that this construction is closely related to equivalence classes in Mazurkiewics trace theory [13], with wellformedness as the dependency relation of the equivalence classes.

*Example 1.* The program from Fig. 1 (Section 2) has the following traces:

$$\begin{aligned}
\tau_1 &= \mathit{new}\langle o_{\mathtt{main}}, o\rangle \cdot \mathit{inv}\langle o_{\mathtt{main}}, f_{\mathtt{m1}}\rangle \cdot \mathit{inv}\langle o_{\mathtt{main}}, f_{\mathtt{m2}}\rangle \cdot \mathit{sched}\langle o, f_{\mathtt{m1}}\rangle \cdot \mathit{sched}\langle o, f_{\mathtt{m2}}\rangle\\
\tau_2 &= \mathit{new}\langle o_{\mathtt{main}}, o\rangle \cdot \mathit{inv}\langle o_{\mathtt{main}}, f_{\mathtt{m1}}\rangle \cdot \mathit{sched}\langle o, f_{\mathtt{m1}}\rangle \cdot \mathit{inv}\langle o_{\mathtt{main}}, f_{\mathtt{m2}}\rangle \cdot \mathit{sched}\langle o, f_{\mathtt{m2}}\rangle\\
\tau_3 &= \mathit{new}\langle o_{\mathtt{main}}, o\rangle \cdot \mathit{inv}\langle o_{\mathtt{main}}, f_{\mathtt{m1}}\rangle \cdot \mathit{inv}\langle o_{\mathtt{main}}, f_{\mathtt{m2}}\rangle \cdot \mathit{sched}\langle o, f_{\mathtt{m2}}\rangle \cdot \mathit{sched}\langle o, f_{\mathtt{m1}}\rangle
\end{aligned}$$

Observe that traces $\tau_1$ and $\tau_2$ belong to the same global trace set (i.e. $[\tau_1] = [\tau_2]$), and will produce the same final state.

Let $G \overset{o:f}{\Longrightarrow} G'$ denote a run between consecutive stable configurations which executes the process identified by $f$ on object $o$ in the stable configuration $G$ until the next stable configuration $G'$. If $\mathit{sched}\langle o, f\rangle \cdot \tau$ is the trace of $G \overset{o:f}{\Longrightarrow} G'$, then $\tau$ is a trace over the event set $\{\mathit{inv}\langle o, f'\rangle,\ \mathit{new}\langle o, o'\rangle,\ \mathit{futWr}\langle o, f\rangle \mid o', f' \in \mathit{Identifiers}\}$. This observation provides an intuition for the following lemma:

**Lemma 2 (Local confluence).** *Let* $G_1, G_2, G_3$ *be stable configurations,* $o, o'$ *object and* $f, f'$ *future identifiers, with* $o \neq o'$, $f \neq f'$. *If* $G_1 \overset{o:f}{\Longrightarrow} G_2$ *and* $G_1 \overset{o':f'}{\Longrightarrow} G_3$, *then there is a stable configuration* $G_4$ *such that* $G_2 \overset{o':f'}{\Longrightarrow} G_4$ *and* $G_3 \overset{o:f}{\Longrightarrow} G_4$.

*Proof (sketch).* The proof follows from the fact that execution in an object does not inhibit a process to run in another object. □

The following theorem shows that local confluence implies global confluence for executions in the *same global trace set* (which means that the two executions agree on the local trace projections).

**Theorem 1 (Global confluence).** *Let* $G_1, G_2, G_3$ *be stable configurations and* $\tau_1, \tau_2$ *traces such that* $G_1 \overset{\tau_1}{\Rightarrow} G_2$ *and* $G_1 \overset{\tau_2}{\Rightarrow} G_3$*. If* $\tau_2 \in [\tau_1]$ *then* $G_2 = G_3$*.*

*Proof (sketch).* Observe that runs with traces in the same global trace set must agree on the naming of objects and futures. The result then follows by induction over the length of $G_1 \overset{\tau_1}{\Rightarrow} G_2$ from local confluence (Lemma 2). □

### 4 Global Reproducibility with Local Traces

The global confluence of executions with traces in the same global trace set provides a formal justification for a method to obtain global reproducibility for distributed active object systems which exhibit non-deterministic behavior. The method is based on enforcing the local trace projection from the global trace set on each active object. For the basic active object language, the method is based on recording the events from the set $\mathcal{E}$ during an execution. This set of events, which includes events capturing the scheduling decisions of the runtime system as well as the choice of dynamically created names during a particular execution, is sufficient to establish the wellformedness of the recorded trace and identify the global trace set of the recorded run. Furthermore, if we record local traces for each active object, these will correspond to the local trace projections of the global trace set. In fact, any composition of local traces recorded during a run will result in the same global trace set. Similarly, any composition of local trace projections enforced during a replay will result in a trace in the same global trace set. Thus, Theorem 1 guarantees that local recording and replay of different traces from the same global trace set will result in the same final state. It remains to show that for any such trace in the global trace set corresponding to a recorded run, the execution during replay will not get stuck. For this purpose, we now formalize record and replay as extensions to the semantics of the basic active object language.

We extend the operational semantics of Fig. 6 to record and replay traces. Let $\tau \rhd cn$ denote an extended runtime configuration, where $\tau$ is a witness for $cn$, playing dual roles for recording and replaying. A *recorded run* starts from an initial configuration $\epsilon \rhd cn$, where $cn$ is the initial configuration of the run to be recorded. The reduction system for recording a trace is given as a relation $\overset{\bullet}{\longrightarrow}$ by the rules in Fig. 7; the two rules correspond to the unlabeled (just Global-Load) and labeled transitions of the semantics, respectively. A replay starts from an initial configuration $\tau \rhd cn$, where $\tau$ is a trace and $cn$ the initial configuration of the run to be replayed. The reduction system for replaying a trace is given as a relation $\overset{\blacktriangleright}{\longrightarrow}$ by the rules in Fig. 8, the two rules are symmetric to those for recording a run. The rules in Fig. 7 and Fig. 8 formalize the obvious relation between the recording and replaying of a trace and a run in the semantics of the

$$\frac{cn \to cn'}{\tau \rhd cn \xrightarrow{\bullet} \tau \rhd cn'} \ (\textsc{Unlabeled-Record})$$

$$\frac{cn \xrightarrow{ev} cn'}{\tau \rhd cn \xrightarrow{\bullet} \tau \cdot ev \rhd cn'} \ (\textsc{Labeled-Record})$$

Fig. 7: Semantics of Record

$$\frac{cn \to cn'}{\tau \rhd cn \xrightarrow{\blacktriangleright} \tau \rhd cn'} \ (\textsc{Unlabeled-Replay})$$

$$\frac{cn \xrightarrow{ev} cn'}{ev \cdot \tau \rhd cn \xrightarrow{\blacktriangleright} \tau \rhd cn'} \ (\textsc{Labeled-Replay})$$

Fig. 8: Semantics of Replay basic active object language. Let $\overset{\bullet}{\Longrightarrow}$ and $\overset{\blacktriangleright}{\Longrightarrow}$ denote the reflexive, transitive closures of $\xrightarrow{\bullet}$ and $\xrightarrow{\blacktriangleright}$, respectively.

**Lemma 3 (Freshness of names).** *For any recording $\epsilon \rhd cn \overset{\bullet}{\Longrightarrow} \tau \rhd cn'$ or replay $\tau \cdot \tau' \rhd cn \overset{\blacktriangleright}{\Longrightarrow} \tau' \rhd cn'$, we have that $names(\tau) = names(cn')$.*

*Proof (sketch).* Follows by induction over the length of $\epsilon \rhd cn \overset{\bullet}{\Longrightarrow} \tau \rhd cn'$ and $\tau \cdot \tau' \rhd cn \overset{\blacktriangleright}{\Longrightarrow} \tau' \rhd cn'$, respectively. □

It follows from Lemma 3 that given an identifier $x \in Identifiers$ and a run $\epsilon \rhd cn \overset{\bullet}{\Longrightarrow} \tau \rhd cn'$, if $x \notin names(\tau)$, then $x \notin names(cn')$ and consequently, the predicate *fresh*$(x)$ will hold as a premise for any rule in the semantics that one may want to apply to $cn'$. Consequently, fresh-predicates in the premises of the transition rules of the basic active language will accept the identifier names chosen from the recorded trace when replaying a run.

**Lemma 4 (Progress for replay by global trace).** *Let $G, G'$ be stable configurations. If $\epsilon \rhd G \overset{\bullet}{\Longrightarrow} \tau \rhd G'$ then $\tau \rhd G \overset{\blacktriangleright}{\Longrightarrow} \epsilon \rhd G'$.*

*Proof.* The proof is by induction over the length of the run $\epsilon \rhd G \overset{\bullet}{\Longrightarrow} \tau \rhd G'$. The base case is obvious. We assume (IH) that if $\epsilon \rhd G \overset{\bullet}{\Longrightarrow} \tau \rhd cn$ then $\tau \rhd G \overset{\blacktriangleright}{\Longrightarrow} \epsilon \rhd cn$ and show that if $\epsilon \rhd G \xrightarrow{\bullet} \tau \cdot ev \rhd cn'$ then $\tau \cdot ev \rhd G \xrightarrow{\blacktriangleright} \tau \rhd cn'$. By the IH, this amounts to showing that if $\epsilon \rhd cn \xrightarrow{\bullet} \epsilon \cdot ev \rhd cn'$ then $ev \cdot \epsilon \rhd cn \xrightarrow{\blacktriangleright} \epsilon \rhd cn'$. The proof proceeds by cases over the transition rules of the basic active object language (cf. Fig. 5). The interesting cases are the rules which need new names. Lemma 3 ensures that the predicate *fresh*$(o)$ will hold for a new name $o$ in $ev$ (and similarly for $f$), and the corresponding rules can be applied. □

It follows from Theorem 1 that if we can replay a run which is equivalent to a recorded run $\tau$, the final state of the replayed run will be the same as for the recorded run. It remains to show that any run in the equivalence class $[\tau]$ can in fact be replayed.

**Theorem 2 (Progress for replay by local control).** *Let $G, G'$ be stable configurations, $\tau, \tau'$ traces. If $\epsilon \rhd G \overset{\bullet}{\Longrightarrow} \tau \rhd G'$ and $\tau' \in [\tau]$, then $\tau' \rhd G \overset{\blacktriangleright}{\Longrightarrow} \epsilon \rhd G'$.*

*Proof (sketch).* We show by induction over the length of trace $\tau$ that if $\epsilon \rhd G \overset{\bullet}{\Longrightarrow} \tau \rhd cn$ and $\tau' \in [\tau]$, then $\tau' \rhd G \overset{\blacktriangleright}{\Longrightarrow} \epsilon \rhd cn'$. It then follows from Theorem 1 that $cn = cn'$. □

### 5 Extensions for Richer Active Object Languages

The method for global reproducibility of executions for a basic active object language based on record & replay of local traces, may be extended to include features introducing other sources of non-determinism in richer active object languages [2]. We here briefly review some such features and how the method may be extended to cover them.

*Cooperative scheduling.* In cooperatively scheduled languages (e.g., [3–5, 14]), methods may explicitly *release control*, allowing other pending method invocations be scheduled. The criteria for being rescheduled may be that some boolean condition is met, or a future being resolved. Note that methods still execute until it cooperatively releases control; i.e., a method will not be interrupted because the condition of another method is satisfied. With cooperative scheduling, the same task may be scheduled several times, which means that the same scheduling event may occur multiple times in a trace. In the method for reproducibility, this extension can be covered by an additional suspension-event reflecting the processor release and an adjustment of the wellformedness condition to reflect that a scheduling event either comes after a invocation event (as for the basic language) or after a suspension event on the same future.

*Concurrent object groups.* In language with concurrent object groups (e.g., [3,5]), a group of concurrent objects (or cog) share a common scheduler, which becomes the unit of distribution; this gives an interleaved semantics between objects within the same cog, while separate cogs are truly concurrent. For record & replay, the events of a trace need to capture the cog, rather than the object, in which an event originated. Recording the names of cogs is sufficient for reproducibility without controlling the naming of objects. For the reproducibility method, the proofs in Section 4 would use an equivalence relation between configurations that only differ in the choice of object names inside the cogs and the global trace set (Def. 4) would project on cogs rather than objects.

*Resource-aware behavior.* Active objects may reside in a resource center with limited resources, e.g. CPU or memory restrictions, with regards to time (e.g., [6, 15]). Statements may have some associated cost which requires available resources in order to execute. If there are insufficient resources, then execution is blocked in that object until time advances. Here, object compete for resources, in the same sense that tasks compete for processing time. Following our method for deterministic replay, the traces can be extended with events for resource request in a similar manner as method invocations in the basic active objects language, and resource provision with events similar to the task scheduling events.

*External non-determinism and random numbers.* Active object languages may also feature external factors that may influence an execution, such as input from a user, fetching data from a database or receiving input from a socket, or random number generation. Here, a purely *ordering-based* method is insufficient. Our replay method needs to be extended with events which include the data received from the external source and the replay would need to fetch data from the trace rather than from the external source, similar to the reuse of object and future identifiers from the trace in the previous section. Random number generation can be seen as a special case of external non-determinism; for pseudo-random number generators it would be sufficient to only record the initial seed for reuse during replay.

### 6 Implementing Record & Replay for Real-Time ABS

We report on our implementation[1] of *record & replay*, based on the formalization in Section 4. The implementation was done for Real-Time ABS [6,16], an active object modeling language which includes the following features discussed in Section 5: cooperative scheduling, concurrent object groups, and timed, resource-aware behavior, all of which are handled by our implementation. The simulator for Real-Time ABS models, written in Erlang, supports interaction with a model during execution via the Model API [17] in order to, e.g. fetch the current state of an object, advance the simulated clock or visualize the resource consumption of a running model. In addition, we have implemented a *visualizer* for recorded traces. In this section, we discuss the following aspects of the implementation: the recording of traces in a distributed setting, the handling of names, the visualization of traces, and performance characteristics for the implementation of record & replay.

#### 6.1 Recording Traces in a Distributed Setting

For simulation, ABS models are transpiled to Erlang code by representing most entities as Erlang actors, e.g., concurrent object groups (or cogs), resource centers, futures and ABS-level processes. Thus, execution is concurrent and may be distributed over multiple machines. This leads to two important differences from the formalization in Section 4:

– *True concurrency:* The formalization is based on an interleaved concurrency model, which yields a total order of events. In the simulator, cogs are implemented as Erlang actors and may operate in true parallel, where two events may happen *simultaneously*, which corresponds to a partial order of events.
– *Distributed state:* Because the state of the model is distributed over many independent actors, we cannot easily synchronize over the state of different actors. In the implementation, such synchronization in the formalization must be realized by asynchronous message passing protocols.

[1] The Real-Time ABS simulator is available at
https://github.com/abstools/abstools
The accompanying visualization tool is available at
https://github.com/larstvei/ABS-traces

These differences pose challenges for recording and replaying global traces in the implementation. When recording a run, it is not trivial to obtain a global trace. If all cogs and resource centers were to report their recorded events to a single actor maintaining the global trace, races could occur between different asynchronous messages. For example, if an object $o$ invokes a method on another object $o'$, then the corresponding invocation and scheduling events could arrive in any order. Such races could be resolved by, e.g., introducing additional synchronization or using Lamport timestamps [18, 19]. Similarly, precisely replaying a global trace would require some synchronization protocol with the actor holding the global trace, severely increasing the level of synchronization during execution.

We address these challenges by only considering the local projections of the global trace for each cog and resource center. The information needed to construct local traces does not require any additional synchronization. During replay, only the local execution of an actor is controlled, which is sufficient to obtain a run with a trace in the same global trace set.

#### 6.2 Names in the Erlang Simulator

The formalization allows recorded names to be reused when replaying a run. In contrast, in the Erlang system cogs, resource centers and futures are implemented as actors (i.e. Erlang actors) and identified by a process identifier (pid) determined by Erlang. To ensure that names in the events of the recorded trace are easily identifiable in a replay without modifying the naming scheme of Erlang, we construct additional names that are associated with the given pid. The constructed names follow a deterministic naming scheme, which guarantees that names are globally unique without depending on knowledge of names generated in other actors (in contrast to the fresh-predicate in the semantics).

Cogs, resource centers and futures can be named locally following a naming scheme based on existing actors already having such unique, associated names. The name $\langle A_{id}, i + 1\rangle$ of a new actor can be determined by the actor $A_{id}$ in which it is created, together with a local counter denoting the number $i$ of actors previously created in $A_{id}$. Thus, the name of the actor corresponds to its place in the topology and is guaranteed to be fresh.

#### 6.3 Visualization of Recorded Traces

The trace recorded during a simulation can give the user insight into that execution of a model, since it captures the model's communication structure. The recorded trace may be extracted from a running simulation via the Model API or written to file on termination. However, the terse format of the traces makes it hard for users to quickly get an intuitive idea of what is happening in the model. Complementing the replay facility, we have developed a tool to visualize recorded traces, which conveys information from traces in a more intuitive format. To facilitate visualization, the events in our implementation are slightly richer than those in Definition 2; e.g., they include the name of the method corresponding to the future in the event.

The visualization reconstructs a global trace $\tau$ from its local projections. Since the local ordering of events is already preserved by the recorded traces, we only need to compose local traces in a way that preserves wellformedness. We derive a happens-before relation $\prec$ from wellformedness (Definition 3), and denote its transitive closure by $\lll$.

The happens-before relation $\lll$ gives a partial order of events. In the visualization of the trace $\tau$, all events are depicted by a colored dot. For any two events $e_1, e_2$, $e_1$ is drawn above $e_2$ if $e_1 \lll e_2$; the events are drawn in the same column only if they reside in the same cog or resource center. An arrow is drawn between any two events $e_1, e_2$ if $e_1 \prec e_2$. Events that are independent (i.e., neither $e_1 \lll e_2$ nor $e_2 \lll e_1$) may be drawn in the same row. Events with the same future as argument are drawn with the same color. The tool additionally supports simple navigation in the trace, gives visual indicators of simulated time steps, and supports time advancement in a running model through the model API, making it easy to step forward in time. Fig. 2 illustrates the visualization for two runs of the motivating example.

#### 6.4 Example

Consider a Real-Time ABS model of an image rendering service which can process either still photos or video. The service is modeled as a class `Service` with two methods `photo_request` and `video_request`. The model captures *resource-sensitive behavior* in terms of cost annotations associated with the execution of `skip`-statements inside the two methods and in terms of deadlines provided to each method call. The processing cost for rendering an image is constant (here, the cost is given by the field `image_cost`), but the processing cost of rendering a video depends on the number of frames (captured by a parameter `n` to the method `video_request`). The success of each method call depends on whether it succeeded in processing its job, as specified by the cost annotation, before its deadline passes; this is captured by the expression in the return statement `return (Duration(0) < deadline())`. Remark that `deadline()` is a predefined read-only variable in Real-Time ABS processes. Its value is given by the caller.

In the main block, a server is created on which the service can run. This server is a resource-center with limited processing capacity (called a deployment component in Real-Time ABS [6]), restricting the amount of computation that can happen on the server per time interval in the execution of the model. The service is then deployed on the server (by an annotation `[DC: server]` to `new`-statement. We let a class `Client` (omitted here) model a given number of processing requests to the image rendering service in terms of asynchronously calling the two methods a given number of times (e.g., the call to `video_request` takes the form `[Deadline: Duration(10)] f = s!video_request(n)`, pushing the associated futures `f` to a list, and then counting the number of successful requests when the corresponding futures have been resolved. It is easy to see that the success of calls to the `video_request` method which requires more resources, may depend on whether it is scheduled before or after calls to `photo_request`,

```
class Service {
 Int image_cost = 1;

Bool photo_request() {
  [Cost: image_cost] skip;
  return (Duration(0)
          < deadline());
 }

Bool video_request(Int n) {
  [Cost: n*image_cost] skip;
  return (Duration(0)
          < deadline());
 }
}

// Main block
{
  DC server
   = new DC("Server", 2);
  [DC: server] Service s1
   = new Service();
  new Client(s1, 1, 100);
}
```

Fig. 9: Real-Time ABS code for the photo rendering service.

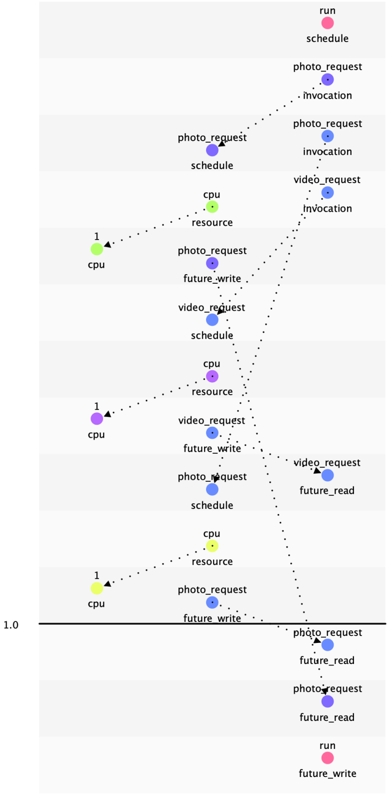

Fig. 10: Visualization of a run of the photo rendering service.

depending on the provided deadlines. Thus, the model exhibits both scheduling non-determinism for asynchronous calls and resource-aware behavior. The image in Fig. 10 depicts a trace from a simulation of the model, showing interactions between a deployment component (left), the service (middle) and the client (right).

#### 6.5 Performance Characteristics of the Implementation

We give a brief evaluation of the performance characteristics of record & replay for Real Time ABS. The size of the traces is proportional to the number of objects, method invocations and resource provisions. Because we do not impose additional synchronization, we are able to achieve a constant-time overhead. To investigate how record & replay scales, we created a micro-benchmark performing method invocations on an active object, and recorded execution times for

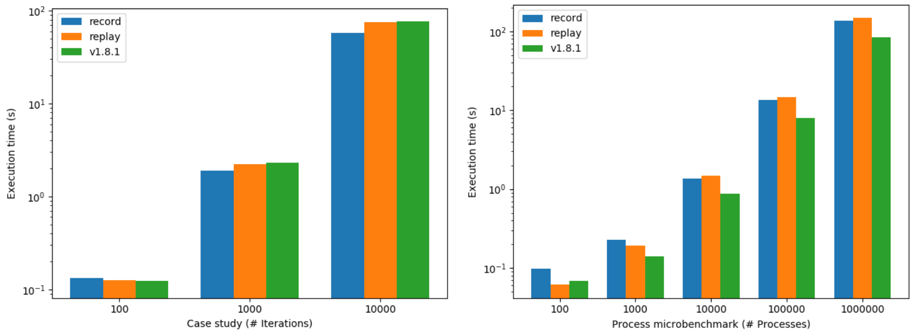

Fig. 11: Record and replay: example (left) and process microbenchmark (right)

$10^2, 10^3, \ldots, 10^6$ method calls. We also ran the example of Section 6.4, recording execution times for $10^2, 10^3$ and $10^4$ Client iterations. These are worst-case scenarios for record & replay, as the invoked methods do not perform any computation that does not result in creating an event.

Fig. 11 shows the results of the two programs with replay enabled, with record enabled and the last release of Real-Time ABS which does not feature record & replay. Note that we only measure simulation time and do not include the time reading and writing trace files. We can see that the results of Fig. 11 (left) are slightly improved and the overhead observed in Fig. 11 (right) is about a factor of 1.8. We note that supporting record & replay in Real-Time ABS required extensive modifications to the Real-Time ABS simulators implementation.

## 7 Related Work

This work complements other analysis techniques for Real-Time ABS models, such as simulation [17], deductive verification [9], and parallel cost analysis [20] and testing [21]. We here discuss related work on deterministic replay. Deterministic replay is an emerging technique to reproduce executions of computer programs in the presence of different non-deterministic factors [1]. It enables cyclic debugging [22] in non-deterministic execution environments. Our focus is on software-level reproducibility in the context of actor-systems. Approaches to reproduce specific runs of non-deterministic systems can be either *content-based* or *ordering based* [23].

*Content-based* methods trace the values read from a shared memory location. These are particularly suitable when there is a lot of external non-determinism (typically I/O operations, like user input). Content-based replay for actor systems typically record messages, including the sender, receiver and message content, (see, e.g., [24–26]). This technique is typically used for rich debuggers like Actoverse [24] for Scala's Akka library, which provides visualization support similar to ours. However, content-based approaches do not scale well [27], because the traces can become very large for message-intensive applications.

*Ordering-based* (or control-based) methods trace a system's *control-flow*. Our work fits within this category. Without external non-determinism, replaying the control-flow will reproduce the data of the recorded run. Ordering-based methods exist for asynchronous message passing using the message passing interface (MPI) [19,28]. MPI assumes that messages from the same source are received in order, this does not generally hold for actor systems. Aumayr *et al.* in [27] study ordering-based replay for actor systems with a memory-efficient representation of the generated traces. Netzer *et al.* [29] propose an interesting method to only trace events directly related to races, rather than all events (removing up to 99% of the events). This line of work is complementary to our focus on formal correctness and low runtime-overhead during record and replay. We believe we could benefit from their work to obtain more efficient trace representations. Lanese *et al.* recently proposed a notion of causal-consistent replay based on reversible debugging [30], which enables replay to a state by only replaying its causal dependencies. Similar to our work, they also formalize record & replay for an actor language. In contrast to our work, their approach is based on a centralized actor for tracing, and can only be used in combination with a debugger [31].

### 8 Conclusion and Future Work

This paper has introduced a method for global reproducibility for runs of distributed Active Object systems, based on local control. The proposed method is order-based and decentralized in that local traces are recorded and replayed without incurring any additional synchronization at the global level. The method is formalized as an operational semantics for a basic active object language, with trace recording and replay. This system exhibits non-determinism through the scheduling of asynchronous method calls and synchronization using first-class futures. Based on this formalization, we justify in terms of properties of trace equivalence classes that local control suffices to reproduce runs with a final state which is equivalent to the final state of a recorded run. We then discuss how other features of active object languages which introduce additional non-determinism can be supported by our method, including cooperative concurrency, concurrent object groups and resource-aware behavior.

The proposed method has been implemented for Real-Time ABS, an Active Object modeling language which includes most of the above-mentioned features and which has a simulator written in Erlang. The implementation only records local ordering information, which allows the overhead of both the record and replay phases to be kept low compared to deterministic replay systems which reproduce an exact global run.

In future work, we plan to build on the proposed record & replay tool for systematic model exploration, by modifying traces between the record and replay phase to explore different runs. This can be done by means of DPOR-algorithms for actor-based systems [32–34]. Combining DPOR with our proposed tool for record & replay would result in a stateless model checker [35] for Active Object systems.

## References

[1] Chen, Y., Zhang, S., Guo, Q., Li, L., Wu, R., Chen, T.: Deterministic replay: A survey. ACM Comput. Surv. **48**(2) (September 2015) 17:1–17:47

[2] de Boer, F., Serbanescu, V., Hähnle, R., Henrio, L., Rochas, J., Din, C.C., Johnsen, E.B., Sirjani, M., Khamespanah, E., Fernandez-Reyes, K., Yang, A.M.: A survey of active object languages. ACM Comput. Surv. **50**(5) (October 2017) 76:1–76:39

[3] Johnsen, E.B., Hähnle, R., Schäfer, J., Schlatte, R., Steffen, M.: ABS: A core language for abstract behavioral specification. In Aichernig, B., de Boer, F.S., Bonsangue, M.M., eds.: Proc. 9th Intl. Symp. on Formal Methods for Components and Objects (FMCO 2010). Volume 6957 of Lecture Notes in Computer Science., Springer (2011) 142–164

[4] Brandauer, S., Castegren, E., Clarke, D., Fernandez-Reyes, K., Johnsen, E.B., Pun, K.I., Tapia Tarifa, S.L., Wrigstad, T., Yang, A.M.: Parallel objects for multicores: A glimpse at the parallel language encore. In: Formal Methods for Multicore Programming (SFM 2015). Volume 9104 of Lecture Notes in Computer Science., Springer (2015) 1–56

[5] Schäfer, J., Poetzsch-Heffter, A.: JCoBox: Generalizing active objects to concurrent components. In D’Hondt, T., ed.: Proc. 24th European Conference on Object-Oriented Programming (ECOOP 2010). Volume 6183 of Lecture Notes in Computer Science., Springer (2010) 275–299

[6] Johnsen, E.B., Schlatte, R., Tapia Tarifa, S.L.: Integrating deployment architectures and resource consumption in timed object-oriented models. J. Log. Algebr. Meth. Program. **84**(1) (2015) 67–91

[7] Albert, E., de Boer, F.S., Hähnle, R., Johnsen, E.B., Schlatte, R., Tapia Tarifa, S.L., Wong, P.Y.H.: Formal modeling and analysis of resource management for cloud architectures: an industrial case study using real-time ABS. Service Oriented Computing and Applications **8**(4) (2014) 323–339

[8] Kamburjan, E., Hähnle, R., Schön, S.: Formal modeling and analysis of railway operations with active objects. Sci. Comput. Program. **166** (2018) 167–193

[9] Din, C.C., Tapia Tarifa, S.L., Hähnle, R., Johnsen, E.B.: History-based specification and verification of scalable concurrent and distributed systems. In: Proc. 17th Intl. Conf. on Formal Engineering Methods (ICFEM 2015). Volume 9407 of Lecture Notes in Computer Science., Springer (2015) 217–233

[10] Bezirgiannis, N., de Boer, F.S., Johnsen, E.B., Pun, K.I., Tapia Tarifa, S.L.: Implementing SOS with active objects: A case study of a multicore memory system. In: Proc. 22nd Intl. Conf. on Fundamental Approaches to Software Engineering (FASE 2019). Volume 11424 of Lecture Notes in Computer Science., Springer (2019) 332–350

[11] Armstrong, J.: Programming Erlang: Software for a Concurrent World. Pragmatic Bookshelf Series. Pragmatic Bookshelf (2007)

[12] Din, C.C., Owe, O.: A sound and complete reasoning system for asynchronous communication with shared futures. J. Log. Algebr. Meth. Program. **83**(5-6) (2014) 360–383

[13] Mazurkiewicz, A.W.: Trace theory. In Brauer, W., Reisig, W., Rozenberg, G., eds.: Advances in Petri Nets 1986. Volume 255 of Lecture Notes in Computer Science., Springer (1987) 279–324

[14] Johnsen, E.B., Owe, O.: An asynchronous communication model for distributed concurrent objects. Software and Systems Modeling **6**(1) (2007) 39–58

[15] Albert, E., Genaim, S., Gómez-Zamalloa, M., Johnsen, E.B., Schlatte, R., Tapia Tarifa, S.L.: Simulating concurrent behaviors with worst-case cost bounds. In Butler, M.J., Schulte, W., eds.: Proc. 17th International Symposium on Formal Methods (FM 2011). Volume 6664 of Lecture Notes in Computer Science., Springer (2011) 353–368

[16] Bjørk, J., de Boer, F.S., Johnsen, E.B., Schlatte, R., Tapia Tarifa, S.L.: User-defined schedulers for real-time concurrent objects. Innovations in Systems and Software Engineering **9**(1) (2013) 29–43

[17] Schlatte, R., Johnsen, E.B., Mauro, J., Tapia Tarifa, S.L., Yu, I.C.: Release the beasts: When formal methods meet real world data. In: It’s All About Coordination - Essays to Celebrate the Lifelong Scientific Achievements of Farhad Arbab. Volume 10865 of Lecture Notes in Computer Science., Springer (2018) 107–121

[18] Lamport, L.: Time, clocks, and the ordering of events in a distributed system. Commun. ACM **21**(7) (1978) 558–565

[19] Ronsse, M., Kranzlmüller, D.: Rolt$${}^{\text{mp}}$$-replay of Lamport timestamps for message passing systems. In: Proc. 6th Euromicro Workshop on Parallel and Distributed Processing (PDP’98), IEEE (1998) 87–93

[20] Albert, E., Correas, J., Johnsen, E.B., Pun, V.K.I., Román-Díez, G.: Parallel cost analysis. ACM Trans. Comput. Log. **19**(4) (2018) 31:1–31:37

[21] Albert, E., Gómez-Zamalloa, M., Isabel, M.: SYCO: a systematic testing tool for concurrent objects. In Zaks, A., Hermenegildo, M.V., eds.: Proc. 25th Intl. Conf. on Compiler Construction (CC 2016), ACM (2016) 269–270

[22] LeBlanc, T.J., Mellor-Crummey, J.M.: Debugging parallel programs with instant replay. IEEE Trans. Computers **36**(4) (1987) 471–482

[23] Ronsse, M., Bosschere, K.D., de Kergommeaux, J.C.: Execution replay and debugging. In: AADEBUG. (2000)

[24] Shibanai, K., Watanabe, T.: Actoverse: a reversible debugger for actors. In Koster, J.D., Bergenti, F., eds.: Proc. 7th Intl. Workshop on Programming Based on Actors, Agents, and Decentralized Control (AGERE 2017), ACM (2017) 50–57

[25] Barr, E.T., Marron, M., Maurer, E., Moseley, D., Seth, G.: Time-travel debugging for javascript/node.js. In Zimmermann, T., Cleland-Huang, J., Su, Z., eds.: Proc. 24th Intl. Symp. on Foundations of Software Engineering (FSE 2016), ACM (2016) 1003–1007

[26] Burg, B., Bailey, R., Ko, A.J., Ernst, M.D.: Interactive record/replay for web application debugging. In Izadi, S., Quigley, A.J., Poupyrev, I., Igarashi, T., eds.: Proc. 26th Symp. on User Interface Software and Technology (UIST’13), ACM (2013) 473–484

[27] Aumayr, D., Marr, S., Béra, C., Boix, E.G., Mössenböck, H.: Efficient and deterministic record & replay for actor languages. In Tilevich, E., Mössenböck, H., eds.: Proc. 15th Intl. Conf. on Managed Languages & Runtimes (ManLang’18), ACM (2018) 15:1–15:14

[28] de Kergommeaux, J.C., Ronsse, M., Bosschere, K.D.: MPL*: Efficient record/play of nondeterministic features of message passing libraries. In Dongarra, J.J., Luque, E., Margalef, T., eds.: Recent Advances in Parallel Virtual Machine and Message Passing Interface, proc. 6th European PVM/MPI Users’ Group Meeting. Volume 1697 of Lecture Notes in Computer Science., Springer (1999) 141–148

[29] Netzer, R.H.B., Miller, B.P.: Optimal tracing and replay for debugging message-passing parallel programs. The Journal of Supercomputing **8**(4) (1995) 371–388

[30] Lanese, I., Palacios, A., Vidal, G.: Causal-consistent replay debugging for message passing programs. In Pérez, J.A., Yoshida, N., eds.: Proc. 39th Intl. Conf. on Formal Techniques for Distributed Objects, Components, and Systems (FORTE 2019). Volume 11535 of Lecture Notes in Computer Science., Springer (2019) 167–184

[31] Lanese, I., Nishida, N., Palacios, A., Vidal, G.: Cauder: A causal-consistent reversible debugger for erlang. In Gallagher, J.P., Sulzmann, M., eds.: Proc. 14th Intl. Symp. on Functional and Logic Programming (FLOPS 2018). Volume 10818 of Lecture Notes in Computer Science., Springer (2018) 247–263

[32] Flanagan, C., Godefroid, P.: Dynamic partial-order reduction for model checking software. In Palsberg, J., Abadi, M., eds.: Proc. 32nd Symp. on Principles of Programming Languages (POPL 2005), ACM (2005) 110–121

[33] Abdulla, P.A., Aronis, S., Jonsson, B., Sagonas, K.: Optimal dynamic partial order reduction. In Jagannathan, S., Sewell, P., eds.: Proc. 41st Symposium on Principles of Programming Languages (POPL’14), ACM (2014) 373–384

[34] Albert, E., Arenas, P., de la Banda, M.G., Gómez-Zamalloa, M., Stuckey, P.J.: Context-sensitive dynamic partial order reduction. In Majumdar, R., Kuncak, V., eds.: Proc. 29th Intl. Conf. on Computer Aided Verification (CAV 2017). Volume 10426 of Lecture Notes in Computer Science., Springer (2017) 526–543

[35] Godefroid, P.: Model checking for programming languages using Verisoft. In Lee, P., Henglein, F., Jones, N.D., eds.: Proc. 24th Symp. on Principles of Programming Languages (POPL 1997), ACM (1997) 174–186

